# Exploring the neural correlates of (altered) moral cognition in psychopaths

**DOI:** 10.1002/bsl.2539

**Published:** 2021-10-15

**Authors:** Laura M. Lenzen, Maximilian R. Donges, Simon B. Eickhoff, Timm B. Poeppl

**Affiliations:** 1 Department of Psychiatry, Psychotherapy and Psychosomatics, Faculty of Medicine, RWTH Aachen University, Aachen, Germany; 2 Department of Psychiatry and Psychotherapy, University of Regensburg, Regensburg, Germany; 3 Institute of Neuroscience and Medicine (INM-7), Brain and Behaviour, Research Centre Jülich, Jülich, Germany; 4 Institute for Systems Neuroscience, Heinrich Heine University, Düsseldorf, Germany

**Keywords:** empathy, meta-analysis, moral cognition, neuroimaging, psychopathy, theory of mind

## Abstract

Research into the neurofunctional mechanisms of psychopathy has gathered momentum over the last years. Previous neuroimaging studies have identified general changes in brain activity of psychopaths. In an exploratory meta-analysis, we here investigated the neural correlates of impaired moral cognition in psychopaths. Our analyses replicated general effects in the dorsomedial prefrontal cortex, lateral prefrontal cortex, fronto-insular cortex, and amygdala, which have been reported recently. In addition, we found aberrant brain activity in the midbrain and inferior parietal cortex. Our preliminary findings suggest that alterations in both regions may represent more specific functional brain changes related to (altered) moral cognition in psychopaths. Furthermore, future studies including a more comprehensive corpus of neuroimaging studies on moral cognition in psychopaths should re-examine this notion.

## 1 | INTRODUCTION

With an incidence of 1%, psychopathy is a phenomenon of considerable importance in the general population (Coid & Yang, 2008). This is not least because psychopaths commit a disproportionate amount of crimes (Lösel, 2001), especially violent acts. Psychopathic offenders are more than twice as likely to reoffend after committing a crime as nonpsychopathic offenders (Mokros et al., 2014). In part because of its association with violence and recidivism, the phenomenon of psychopathy is of high public interest (Hare & Neumann, 2008; Leistico et al., 2008).

Historically, the meaning of the term psychopathy has been modified over time. The American psychiatrist Hervey Cleckley, in his influential 1941 monograph, The Mask of Sanity (Cleckley, 1941), coined the modern concept of psychopathy. According to the criteria established by Cleckley (1941), the central characteristics of psychopathy include pathological egocentrism, an inability to love others, deceitfulness, and a lack of remorse or shame. In the 1970s, the Canadian psychologist Robert D. Hare went on to contribute significantly to the development of the modern concept of psychopathy by designing the most widely used diagnostic tool for psychopathy today, the Psychopathy Checklist (PCL-R; Hare, 2003; Hare & Neumann, 2008). In his concept, Hare (2003) defines a total of 20 characteristics of psychopathy that must be assessed and scored on a 3-point scale. Moreover, the named items can be distinguished into two subtypes. One subset captured interpersonal and affective features of psychopathy, such as superficial affect, inflated self-esteem, pathological lying, or lack of empathy. The second subgroup included symptoms related to antisocial behavior of the individual, such as impulsivity, irresponsibility, low behavioral control, or juvenile delinquency (Hare, 2003). Both Hervey Cleckley and Robert D. Hare highlight the importance of the lack of emotional resonance as a pathognomonic symptom of psychopathy (Cleckley, 1941; Hare & Neumann, 2008).

The American Psychiatric Association (APA, 2013) and the World Health Organization (WHO, 1992) define a lack of anxiety or fear and a bold interpersonal style as the distinguishing characteristics of psychopathy (APA, 2013; WHO, 1992). Using the aforementioned criteria, they differentiate psychopathy from the antisocial personality disorder. Few people with antisocial personality disorder meet the criteria of psychopathy, whereas the other way around it is usually the case. Nevertheless, it is important to emphasize that psychopathy is not exclusively associated with antisocial or dissocial personality disorder. It can equally occur as a comorbidity of borderline or narcissistic personality disorder (Miller et al., 2010; Nioche et al., 2010; Sprague et al., 2012).

What most of the concepts of psychopathy described have in common, is that psychopaths are often denied certain interpersonal qualities. Psychopaths, according to today’s understanding of the term, are indeed capable of establishing superficial relationships. However, they are said to have a high manipulative potential, a lack of empathy, and social responsibility as well as a lack of guilt and remorse (Hare & Neumann, 2008). These characteristics could be summarized as an impairment of “moral cognition” in psychopaths.

Psychopaths are thought to have a weak sensitivity to moral norms (Li et al., 2020), which is why morally inappropriate behavior is often considered pathognomonic for them. In addition, there is research suggesting that psychopaths exhibit abnormal emotional profiles and have diminished inhibitory control, which conditions their higher potential for aggression (Glenn & Raine, 2008; Kiehl, 2006; Kiehl et al., 2001; Raine & Yang, 2006). However, there is disagreement about the extent to which the amorality of psychopaths is based on an impairment of specific moral knowledge, as some studies suggest (Blair, 1995, 2008), or whether they are perfectly capable of distinguishing between right and wrong but just do not act accordingly (Cima et al., 2010).

Within the last years, there have been many attempts to identify changes in brain activity associated with psychopathy and how these norm deviations might be related to pathognomonic traits in psychopaths. Many neuroimaging studies from the past have unsuccessfully tried to identify brain abnormalities that might be characteristic of psychopathy (Koenigs et al., 2011; Pujol et al., 2019). However, given the large heterogeneity of sample results, it was considered premature to draw conclusions with regard to neural circuits that might be affected in psychopathy (Koenigs et al., 2011).

A previous meta-analysis summarized existing imaging data on this topic and examined for altered brain activity in psychopaths (Poeppl et al., 2019). Although this study showed generally altered brain activity in psychopaths, the study design did not allow to answer the question of possible, more specific neural correlates for alterations in what is called “moral cognition” in psychopathy. Therefore, the aim of the present study was to summarize the existing neuroimaging data investigating “moral cognition” in psychopaths and to identify alterations in associated neural activity using an automated meta-analysis.

## 2 | METHODS

### 2.1 | Coordinate-based meta-analysis

#### 2.1.1 | Data selection

We used a common procedure to identify relevant experimental studies. In the first step, we selected studies through a standard search in the PubMed (https://www.ncbi.nlm.nih.gov/pubmed/) and ISI Web of Science (https://www.webofknowledge.com) databases using the terms “psychopathy” or “psychopathic” in combination with “fMRI,” “functional MRI,” “functional magnetic resonance,” “PET,” “positron emission,” “ASL,” “arterial spin labeling,” “MEG,” “magnetoencephalography,” “neuroimaging,” or “imaging.” In a second step, further were found by means of the “related articles” function of the PubMed database and by tracing the references from the identified papers and review articles.

We only included experimental studies that investigated effects of psychopathy on neural correlates of morality, empathy or theory of mind, that is, moral cognition (Bzdok et al., 2012). To enable comparison with robust, do-main-unspecific functional brain alterations of psychopaths, we only included experiments that have also been included in a recent meta-analysis of generally aberrant brain activity associated with psychopathy (Poeppl et al., 2019). Neuroimaging experiments that met these criteria were considered relevant when they reported either (1) direct group comparisons between psychopathic and non-psychopathic subjects or (2) correlations of brain activity with an established measure of psychopathy (e.g., the revised Psychopathy Checklist [PCL-R]; Hare, 2003). Both strategies are valid to objectify alterations in brain activity associated with psychopathy because psychopathy can be conceptualized categorically as well as dimensionally (Coid & Yang, 2008). In addition, both approaches related psychopathy to commonly used measures of this disorder. Furthermore, only experiments reporting results of whole-brain group analyses with coordinates referring to a standard reference space (Talairach-Tournoux or Montreal Neurological Institute [MNI]) were included. We excluded results of region-of-interest analyses and studies not reporting stereotaxic coordinates.

On the basis of these search criteria, 45 experiments were found to be eligible for inclusion into the meta-analyses (Table S1 in the Supporting Information). Only fMRI but no PET, ASL, or MEG investigations fulfilled our search criteria. Together, these experiments reported 237 foci (with “experiment” referring to an individual contrast reported in this article; cf., Figure S1 and Table S1 in the Supporting Information).

The number of these foci consisted of 58 activations from 9 direct group comparisons (psychopaths > non-psychopaths) and 21 foci of positive correlations between brain activity and psychopathy scales from 13 analyses as well as 116 deactivations from 10 direct group comparisons (psychopaths < non-psychopaths) and 42 foci of negative correlations between brain activity and psychopathy scales from 13 analyses. Differences in coordinate spaces (Talairach vs. MNI space) were accounted for by transforming coordinates reported in Talairach space into MNI coordinates using a linear transformation (Laird et al., 2010).

First, we evaluated convergence of reported activation foci indicating increased brain activity in psychopaths by pooling direct group comparisons (psychopaths > non-psychopaths) and positive correlational analyses (22 experiments, 79 foci). In the same way (i.e., by pooling group comparisons and correlational analyses), we then tested for convergence of reported deactivation foci (23 experiments, 158 foci).

### 2.1.2 | Activation likelihood estimation

Our statistical analyses were carried out using the activation likelihood estimation (ALE) algorithm for coordinate-based meta-analysis of neuroimaging results (Eickhoff et al., 2012; Turkeltaub et al., 2012). This algorithm identifies areas with a convergence of reported coordinates across experiments, that is, higher than expected from a random spatial association. Extracted foci are handled as centers of 3D Gaussian probability distributions capturing the spatial uncertainty associated with each focus (Eickhoff et al., 2012). The between-subject variance is weighted by the number of participants per study, since larger sample sizes should provide more reliable approximations of the “true” activation effect and should therefore be modeled by more “narrow” Gaussian distributions.

As a next step, the probabilities of all foci reported of a given experiment were combined for each voxel, which yielded a modeled activation (MA) map (Figure S2 in the Supporting Information; Turkeltaub et al., 2012). Convergence across experiments was quantified at each site in the brain on the basis of voxelwise ALE scores (union across these MA maps). To distinguish “true” from random convergence, ALE scores were compared to an empirical null distribution reflecting a random spatial association among all MA maps. The resulting random-effects inference focuses on convergence across studies rather than clustering within a particular study (Eickhoff et al., 2009). This null hypothesis was derived by computing the distribution that would be obtained when sampling a voxel at random from each of the MA maps and taking the union of these values in the same manner as for the (spatially contingent) voxels in the original analysis (Eickhoff et al., 2012). The *p*-value of a “true” ALE score was determined based on the proportion of equal or higher values obtained under the null distribution. The resulting nonparametric *p*-values were then assessed using threshold-free cluster enhancement (TFCE; Smith & Nichols, 2009) to correct for multiple comparisons (*p* < 0.05) and transformed into *z* scores for display (Eickhoff et al., 2012).

For anatomical labeling, we capitalized on cytoarchitectonic maps of the human brain provided by the Statistical Parametric Mapping Anatomy Toolbox (Eickhoff et al., 2005, 2006, 2007). Clusters were thus assigned to the most probable histologically defined area at the respective location.

## 3 | RESULTS

### 3.1 | Coordinate-based meta-analysis

**FIGURE 1:**
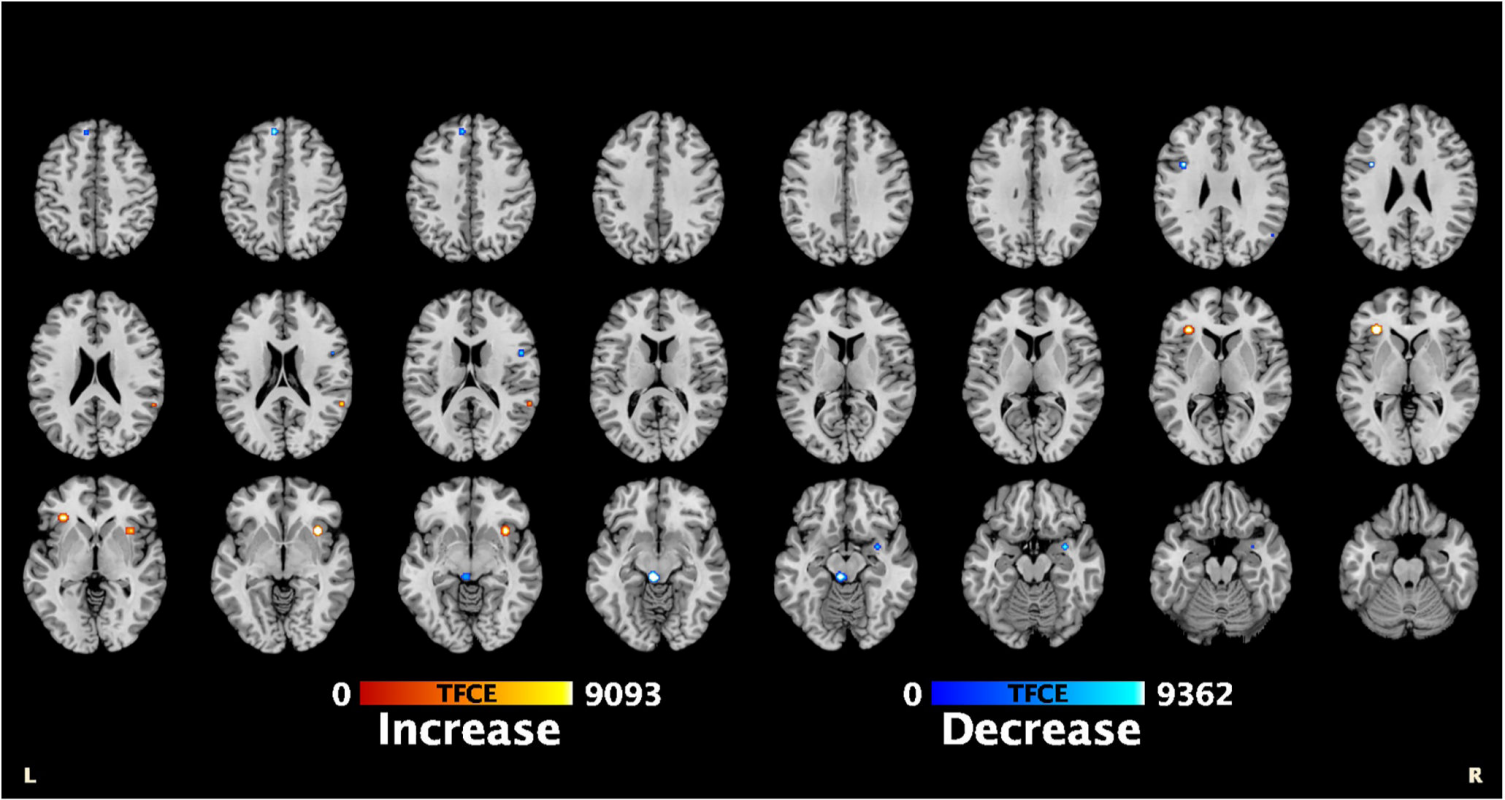
Brain regions associated with moral cognition showing aberrant activity in psychopaths. Significant clusters where the activation likelihood estimation analysis revealed convergence of altered brain activity in corresponding experiments (*p* < 0.05, threshold-free cluster enhancement corrected; cf. Table 1). Orange/blue color indicates in-/decreased activity

Convergence of *increased* neural activity associated with psychopathy was observed in the left fronto-insular and the right insular cortex as well as in the right inferior parietal cortex (Figure 1 and Table 1). In contrast, convergence of *decreased* brain activity in psychopathy was located in the dorsomedial prefrontal cortex, the lateral prefrontal cortex and midbrain on the left hemisphere as well as in the amygdala, the inferior parietal cortex and the lateral prefrontal cortex on the right hemisphere (Figure 1 and Table 1).

Our analyses thus did not identify any region that was not selectively hyper- or hypoactivated. Contribution analyses showed that tasks including moral processing and tasks focusing on empathy contributed to the effects. No specific stimulus or task characteristic thus seemed to critically drive the effects. It can thus not be inferred that certain kinds of design lead to differences in a particular region.

## 4 | DISCUSSION

The prevailing assumption is that the rule-breaking, antisocial behavior of psychopaths is due, at least in part, to brain structural impairments of regions that support moral cognition and emotion (dorsal and ventral prefrontal cortex, amygdala, and angular gyrus; Raine & Yang, 2006). This neurobiological predisposition is certainly only one of several aspects involved in the etiology of psychopathic behavior. Nevertheless, neurobiological findings described raise important neuroethical questions (Raine & Yang, 2006).

In the present meta-analysis, we sought to discern the effects of psychopathy on the functional neural correlates of morality, empathy, and theory of mind, that is, on moral cognition. We sought to identify abnormalities in brain activity to assess if specifically altered activity might be related to the diminished moral cognition pathognomonic in psychopaths. To this end, we performed meta-analyses of whole-brain neuroimaging studies on altered brain activity in psychopaths in paradigms of moral cognition.

Our analyses revealed *decreased* activity in left and right lateral prefrontal cortex, dorsomedial prefrontal cortex, right amygdala, right inferior parietal cortex, and midbrain. In contrast, *increased* activity was found in the right insular cortex, left fronto-insular cortex, and another region of the right inferior parietal cortex. In comparison with a previous meta-analysis on generally aberrant brain activity in psychopaths (Poeppl et al., 2019), the present study identified additional effects in the midbrain and right inferior parietal cortex. Hence, these additional alterations may represent a functional deficit in psychopaths specifically related to moral cognition.

The present meta-analytic data suggest that psychopaths have reduced activity in the dorsomedial prefrontal cortex. This finding is consistent with results from previous meta-analyses (Bzdok et al., 2012; Poeppl et al., 2019). The brain region of the dorsomedial prefrontal cortex has been implicated in social cognition, which includes empathy, morality, and theory of mind. A previous meta-analysis showed that the dorsomedial prefrontal cortex is consistently involved in all of these three domains (Bzdok et al., 2012).

Decreased inhibitory control is considered a deficit characteristic of psychopaths that may contribute to their impulsive behavior (Blair, 2008; Blair & Cipolotti, 2000; Kiehl, 2006). It is known from previous research that psychopaths have reduced action control (Krakowski et al., 2015). Our meta-analytic finding of reduced activity in the right lateral prefrontal cortex, which has been associated with cognitive control and action execution (Poeppl et al., 2019), might be the neurobiological correlate for this phenomenon. If applicable, diminished action control contributes to the morally inappropriate behavior that is pathognomonic for psychopaths (Blair et al., 2002; Nichols, 2002).

The right lateral prefrontal cortex is not only associated with action execution but also with pain processing (Poeppl et al., 2019). Our finding of decreased activity of the right lateral prefrontal cortex is consistent with previous findings suggesting that psychopathy is associated with lower perception of pain (Brislin et al., 2016). Similarly, psychopaths have been shown to feel less empathy for third parties when they appear to be experiencing pain (Decety et al., 2013).

The meta-analysis also revealed decreased activity in the right amygdala, which contributes to emotional reward processing (Poeppl et al., 2019). Emotionless and unemotional behaviors also appear to be associated with decreased amygdala responsiveness in line with other study findings (Blair, 2015). Impaired functional integrity of the amygdala increases the risk for frustration-induced reactive aggression (Blair et al., 2006).

Abnormal activity was found in two regions that have previously been associated with semantic language processing (left fronto-insular cortex and left lateral prefrontal cortex; Poeppl et al., 2019). This observation fits with previous research findings, suggesting that norm deviant processing of semantic and verbal information occurs in psychopaths (Bagley et al., 2009; Kiehl et al., 1999; Williamson et al., 1991).

In addition to these findings that have been reported elsewhere (Poeppl et al., 2019), the present meta-analysis showed decreased activity in the midbrain. A previous meta-analysis on the neural correlates of moral cognition suggested that the midbrain region plays an important role specifically in empathy (Bzdok et al., 2012). In this respect, the altered activity in the midbrain in psychopaths could possibly be related to their reduced empathy. There was also aberrant activity in the right inferior parietal cortex. At first glance it seems puzzling that we observed both increased and decreased activity in nearby regions within the right inferior parietal cortex. However, it matches well with previous findings that this region is significantly involved in all three domains of moral cognition (empathy, moral, and theory of mind) and consists of modules that link antagonistic brain networks (Bzdok et al., 2012, 2013). Both the midbrain and inferior parietal cortex appeared unaltered in previous studies identifying general brain changes in psychopaths (Deming & Koenigs, 2020; Poeppl et al., 2019). Abnormalities in these regions may therefore be specific to altered moral cognition in psychopaths and future studies with a larger data pool should reexamine this hypothesis.

As a limitation of this meta-analysis, it must be noted that not all available functional imaging studies of psychopathy met our inclusion criteria. We excluded studies that restricted their analyses to a limited number of regions (i.e., region-of-interest approaches). In addition, we restricted our analysis to imaging studies that focused on moral cognition in psychopathy and, for the sake of comparability, have also been included in a previous meta-analysis (Poeppl et al., 2019). This allowed us to assess which of the (previously) observed alterations are more general versus more specific to moral cognition. In consequence, however, our results are based on a relatively small number of studies, and not all included experiments were independent from one another. Although contribution analyses showed that the effects were not driven by a single study or task, we thus regard our analyses exploratory and the findings preliminary. Future meta-analyses using the identical approach but including a more comprehensive corpus of neuroimaging studies on moral cognition in psychopaths are needed to provide definite results.

## 5 | CONCLUSION

In summary, the results of this study suggest that altered moral cognition in psychopaths might be related to generally aberrant brain activity in several brain regions but also to more specific alterations in midbrain and inferior parietal cortex. It seems thus possible that this deviance in brain activity in psychopaths is directly related to their psychopathology, that is, their diminished moral cognition. However, the findings of our study do not allow the conclusion on whether the neural alterations are cause or consequence of the disorder and the associated behavioral patterns. The same applies to putative structural brain changes, which may underlie the functional alterations in psychopaths. Alternatively, it is also conceivable that the neuronal differences are just generic effects in polymodal regions of the cortex (Genon et al., 2018).

If replicated in larger meta-analyses, these new insights into the “moral brain” could be used to develop therapeutic strategies for psychopathic behavior. Neuromodulation techniques (e.g., applied over the right inferior parietal cortex) could be used to treat underlying brain dysfunction (Fumagalli & Priori, 2012). The finding that there are neurobiological correlates to deviant behaviors in psychopaths contains neuroethical as well as forensic implications. The incorporation of neuroscientific methods could improve predictive capabilities of recidivism, future dangerousness, and responsiveness in psychopaths (Yoder & Decety, 2018). Also, treatment concepts could be more specifically tailored and, furthermore, the evaluation of the question of culpability in psychopaths would have to be reconsidered (Raine, 2019).

## Supplementary Material

Supplemental Info

## Figures and Tables

**TABLE 1:** Direction of aberrant brain activations related to moral cognition in psychopaths

Direction	Macroanatomical location	Cytoarchitectonic location	Cluster size in voxels	MNI coordinates			TFCE score
				*x*	*y*	*z*	
↑	L fronto-insular cortex	Area Id7	64	−32	30	0	12,123.6
	R insular cortex	Area Id7	52	36	16	−6	11,777.3
	R inferior parietal cortex	Area PGa	12	60	−46	18	7,736.0
↓	L midbrain		54	−4	−30	−12	12,482.3
	L dorsomedial prefrontal cortex		19	−8	40	42	9,273.8
	L lateral prefrontal cortex	Area 44	16	−38	6	26	10,020.2
	R amygdala		12	32	0	−18	9,140.4
	R lateral prefrontal cortex	Area 44	9	50	6	16	9,031.7
	R inferior parietal cortex		1	52	−64	28	8,531.1

*Note*: Convergent increased (↑) and decreased (↓) brain activity related to psychopathy according to ALE across 22 experiments featuring 79 foci (↑) and 23 experiments featuring 158 foci (↓). Results are corrected for multiple comparisons using TFCE (*p* < 0.05).

Abbreviations: ALE, activation likelihood estimation; MNI, Montreal Neurological Institute; TFCE, threshold-free cluster enhancement.
