# Supplementary material for: Exploring the neural correlates of (altered) moral cognition in psychopaths
Source: Behav Sci Law. Author manuscript; Available in PMC 2022 Jun 1. (PMC8688304; doi:10.1002/bsl.2539)
Supplement: Supplemental Info [file NIHMS1753447-supplement-Supplemental_Info.docx]

**Supplementary Material**

**Supplementary Figure 1**

Summary of the 237 activation foci reported in all 45 experiments included in the meta-analyses.


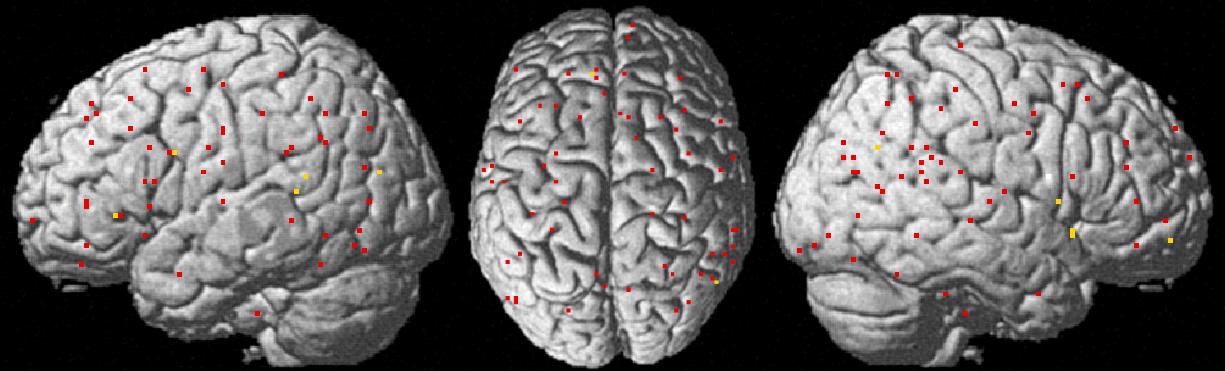


**Supplementary Figure 2**

Activation likelihood estimates (ALE), reflecting, for each voxel, the union of the modeled activation (MA) maps across (a) all experiments reporting aberrant brain activity associated with psychopathy in moral cognition, (b) experiments reporting increased brain activity associated with psychopathy in moral cognition, and (c) experiments reporting decreased brain activity associated with psychopathy in moral cognition.


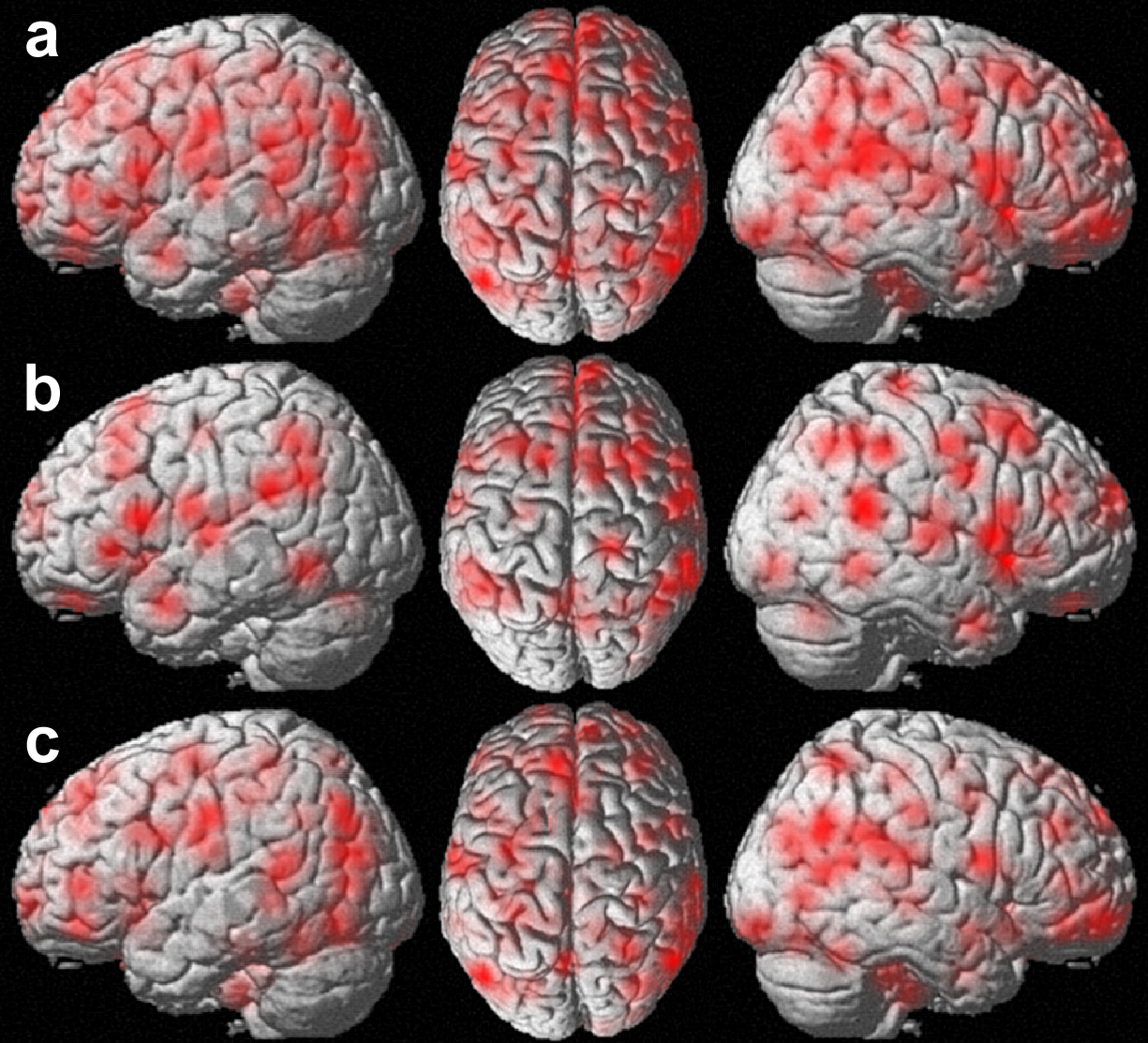


**Supplementary Table**

Studies reporting neuroimaging experiments showing alterations in brain activity associated with psychopathy in moral cognition

| **1^st^ author** | **Year** | **Subjects** | | | **Scale** | **Imaging** | **Task** | **Subject-Level Analysis** | **Group-Level Analysis** | **Foci** | **Direction** |
| --- | --- | --- | --- | --- | --- | --- | --- | --- | --- | --- | --- |
|  |  | Psychopaths | Controls | Others |  |  |  |  |  |  |  |
| Caldwell | 2015 | - | - | 87 | PCL-R | fMRI | Moral processing | Moral > Non-Moral | Correlation w/ PCL-R Factor 1 | 0 | + |
|  |  | - | - | 87 | PCL-R | fMRI | Moral processing | Moral > Non-Moral | Correlation w/ PCL-R Factor 1 | 2 | - |
|  |  | - | - | 87 | PCL-R | fMRI | Moral processing | Moral > Non-Moral | Correlation w/ PCL-R Factor 2 | 0 | + |
|  |  | - | - | 87 | PCL-R | fMRI | Moral processing | Moral > Non-Moral | Correlation w/ PCL-R Factor 2 | 0 | - |
| Decety | 2013 | 24 | 22 | - | PCL-R | fMRI | Empathy processing | Pain Interaction | Controls > Psychopaths | 3 | - |
|  |  | 24 | 22 | - | PCL-R | fMRI | Empathy processing | Pain Interaction | Psychopaths > Controls | 13 | + |
|  |  | 24 | 22 | - | PCL-R | fMRI | Empathy processing | Pain Expression | Controls > Psychopaths | 15 | - |
|  |  | 24 | 22 | - | PCL-R | fMRI | Empathy processing | Pain Expression | Psychopaths > Controls | 2 | + |
|  |  | 24 | 46 | - | PCL-R | fMRI | Empathy processing | Pain Interaction | Correlation w/ PCL-R Factor 1 | 11 | + |
|  |  | 24 | 46 | - | PCL-R | fMRI | Empathy processing | Pain Interaction | Correlation w/ PCL-R Factor 2 | 4 | + |
|  |  | 24 | 46 | - | PCL-R | fMRI | Empathy processing | Pain Interaction | Correlation w/ PCL-R Factor 1 | 2 | - |
|  |  | 24 | 46 | - | PCL-R | fMRI | Empathy processing | Pain Interaction | Correlation w/ PCL-R Factor 2 | 2 | - |
|  |  | 24 | 46 | - | PCL-R | fMRI | Empathy processing | Pain Expression | Correlation w/ PCL-R Factor 1 | 2 | + |
|  |  | 24 | 46 | - | PCL-R | fMRI | Empathy processing | Pain Expression | Correlation w/ PCL-R Factor 2 | 1 | + |
|  |  | 24 | 46 | - | PCL-R | fMRI | Empathy processing | Pain Expression | Correlation w/ PCL-R Factor 1 | 8 | - |
|  |  | 24 | 46 | - | PCL-R | fMRI | Empathy processing | Pain Expression | Correlation w/ PCL-R Factor 2 | 10 | - |
| Harenski | 2010 | 16 | 16 | - | PCL-R | fMRI | Moral processing | N/A | Non-Psychopaths > Psychopaths | 0 | - |
|  |  | 16 | 16 | - | PCL-R | fMRI | Moral processing | N/A | Psychopaths > Non-Psychopaths | 1 | + |
|  |  | 16 | 16 | - | PCL-R | fMRI | Moral processing | Severity of moral violation ratings | Non-Psychopaths > Psychopaths | 2 | - |
|  |  | 16 | 16 | - | PCL-R | fMRI | Moral processing | Severity of moral violation ratings | Psychopaths > Non-Psychopaths | 0 | + |
| Harenski | 2014 | - | - | 157 | PCL-R | fMRI | Moral processing | Moral+Non-Moral > Neutral | Correlation w/ PCL-R | 0 | + |
|  |  | - | - | 157 | PCL-R | fMRI | Moral processing | Moral+Non-Moral > Neutral | Correlation w/ PCL-R | 5 | - |
|  |  | - | - | 157 | PCL-R | fMRI | Moral processing | Moral+Non-Moral > Neutral | Correlation w/ PCL-R Factor 1 | 0 | + |
|  |  | - | - | 157 | PCL-R | fMRI | Moral processing | Moral+Non-Moral > Neutral | Correlation w/ PCL-R Factor 1 | 0 | - |
|  |  | - | - | 157 | PCL-R | fMRI | Moral processing | Moral+Non-Moral > Neutral | Correlation w/ PCL-R Factor 2 | 0 | + |
|  |  | - | - | 157 | PCL-R | fMRI | Moral processing | Moral+Non-Moral > Neutral | Correlation w/ PCL-R Factor 2 | 6 | - |
|  |  | - | - | 157 | PCL-R | fMRI | Moral processing | Moral > Non-Moral | Correlation w/ PCL-R | 0 | + |
|  |  | - | - | 157 | PCL-R | fMRI | Moral processing | Moral > Non-Moral | Correlation w/ PCL-R | 5 | - |
|  |  | - | - | 157 | PCL-R | fMRI | Moral processing | Moral > Non-Moral | Correlation w/ PCL-R Factor 1 | 0 | + |
|  |  | - | - | 157 | PCL-R | fMRI | Moral processing | Moral > Non-Moral | Correlation w/ PCL-R Factor 1 | 1 | - |
|  |  | - | - | 157 | PCL-R | fMRI | Moral processing | Moral > Non-Moral | Correlation w/ PCL-R Factor 2 | 0 | + |
|  |  | - | - | 157 | PCL-R | fMRI | Moral processing | Moral > Non-Moral | Correlation w/ PCL-R Factor 2 | 0 | - |
| Marsh | 2014 | - | - | 16/16 | PPI-R | fMRI | Emotion/Moral processing | Fear > Neutral | Low > High PPI-R Scorers | 1 | - |
|  |  | - | - | 16/16 | PPI-R | fMRI | Emotion/Moral processing | Fear > Neutral | High > Low PPI-R Scorers | 3 | + |
| Meffert | 2013 | 18 | 26 | - | PCL-R | fMRI | Empathy processing | Emotion observation | Controls > Psychopaths | 55 | - |
|  |  | 18 | 26 | - | PCL-R | fMRI | Empathy processing | Emotional empathy | Controls > Psychopaths | 21 | - |
|  |  | 18 | 26 | - | PCL-R | fMRI | Empathy processing | Emotional empathy | Psychopaths > Controls | 33 | + |
| Pujol | 2012 | 22 | 22 | - | PCL-R | fMRI | Moral processing | Congruent > Incongruent | Controls > Psychopaths | 1 | - |
|  |  | 22 | 22 | - | PCL-R | fMRI | Moral processing | Moral Dilemma > Control | Controls > Psychopaths | 5 | - |
| Sommer | 2010 | 14 | 14 | - | PCL-R | fMRI | Theory of Mind | Mentalizing > Reality | Psychopaths > Non-Psychopaths | 1 | + |
|  |  | 14 | 14 | - | PCL-R | fMRI | Theory of Mind | Mentalizing (Unfulfilled) > Reality | Psychopaths > Non-Psychopaths | 2 | + |
| Yoder | 2015 | 28 | 32 | - | PCL-R | fMRI | Moral processing | Bad > Good (Implicit Judgment) | Controls > Psychopaths | 4 | - |
|  |  | 28 | 32 | - | PCL-R | fMRI | Moral processing | Bad > Good (Implicit Judgment) | Psychopaths > Controls | 3 | + |
|  |  | 28 | 32 | - | PCL-R | fMRI | Moral processing | Bad > Good (Implicit Judgment) | Controls > Psychopaths | 10 | - |
|  |  | 28 | 32 | - | PCL-R | fMRI | Moral processing | Bad > Good (Implicit Judgment) | Psychopaths > Controls | 3 | + |

Subjects groups comprised psychopaths and matched controls as well as other samples from the community (Yoder et al., 2015), university community (Marsh et al., 2014), and correctional institutions (Caldwell et al., 2015; Harenski et al., 2014).

CH, Cold Heartedness; CT, Callous Traits; EI, Emotional-Interpersonal; FD, Fearless-Dominance; ; fMRI, functional magnetic resonance imaging; IA, Impulsive-Antisociality; PCL-R, Psychopathy Check List (Hare, 1998); PPI-R, Psychopathic Personality Inventory-Revised (Lilienfeld et al., 2005); SCI, Self-Centered Impulsivity; SD, Socially Deviant.

**Supplementary References**

Caldwell BM, Harenski CL, Harenski KA, Fede SJ, Steele VR, Koenigs MR, Kiehl KA (2015): Abnormal frontostriatal activity in recently abstinent cocaine users during implicit moral processing. Front Hum Neurosci 9:565.

Decety J, Skelly LR, Kiehl KA (2013): Brain response to empathy-eliciting scenarios involving pain in incarcerated individuals with psychopathy. JAMA Psychiatry 70:638–45.

Hare RD (1998): The Hare PCL-R: Some issues concerning its use and misuse. Legal and Criminological Psychology 3:101–22.

Harenski CL, Harenski KA, Shane MS, Kiehl KA (2010): Aberrant neural processing of moral violations in criminal psychopaths. J Abnorm Psychol 119:863–74.

Harenski CL, Edwards BG, Harenski KA, Kiehl KA (2014): Neural correlates of moral and non-moral emotion in female psychopathy. Front Hum Neurosci 8:741.

Lilienfeld SO, Widows MR, Staff PAR (2005): Psychopathic Personality Inventory-Revised. Social Influence 61:97.

Marsh AA, Cardinale EM (2014): When psychopathy impairs moral judgments: neural responses during judgments about causing fear. Soc Cogn Affect Neurosci 9:3–11.

Meffert H, Gazzola V, den Boer JA, Bartels AA, Keysers C (2013): Reduced spontaneous but relatively normal deliberate vicarious representations in psychopathy. Brain 136(Pt 8):2550–62.

Pujol J, Batalla I, Contreras-Rodríguez O, Harrison BJ, Pera V, Hernández-Ribas R, Real E, Bosa L, Soriano-Mas C, Deus J, López-Solà M, Pifarré J, Menchón JM, Cardoner N (2012): Breakdown in the brain network subserving moral judgment in criminal psychopathy. Soc Cogn Affect Neurosci 7:917–23.

Sommer M, Sodian B, Döhnel K, Schwerdtner J, Meinhardt J, Hajak G (2010): In psychopathic patients emotion attribution modulates activity in outcome-related brain areas. Psychiatry Res 182:88–95.

Yoder KJ, Harenski C, Kiehl KA, Decety J (2015b): Neural networks underlying implicit and explicit moral evaluations in psychopathy. Transl Psychiatry 5:e625.
